# *Toxoplasma gondii* infection enhances the kairomonal valence of rat urine

**DOI:** 10.12688/f1000research.3890.1

**Published:** 2014-04-17

**Authors:** Anand Vasudevan, Ajai Vyas

**Affiliations:** 1 School of Biological Sciences, Nanyang Technological University, Singapore, 637551, Singapore

## Abstract

Many animals use chemicals as pheromones to communicate between individuals of the same species, for example to influence mate choice or to assert dominance. Pheromonal communication is an open broadcast system that can be intercepted by unintended receivers such as predators and prey. We have recently reported that male rats infected by the protozoan parasite *Toxoplasma gondii *become more attractive to female rats. This suggests a facilitatory effect of infection on rat pheromone production. In view of the open nature of pheromonal communication, we postulate that *Toxoplasma gondii* infection collateraly enhances kairomonal valence of infected rats to their prey. We compared the strength of kairomonal interception by mice when using scent marks from rats infected with *Toxoplasma gondii* vs. marks from uninfected control rats. Mice exhibited greater avoidance to both fresh urine and aged rat urine marks obtained from infected animals. These results indicate that, at least in some cases, parasitism can result in opportunity costs for hosts by making prey species more averse to them.

## Introduction

The protozoan parasite *Toxoplasma gondii* manipulates the behavior of its rat host in two important ways. First, it not only abolishes the rats’ innate fear of cat odors, it can also induce attraction to cat odors ^1– 4^. This plausibly increases parasite transmission to cats that serve as its definitive hosts. Second, *Toxoplasma gondii* infection enhances the attractiveness of infected males to females ^5^. Interestingly, enhanced attractiveness could benefit both the parasite and its host. This plausibly increases the parasites’ transmission through sexual and vertical routes ^5– 8^ and benefits the hosts by increasing their reproductive opportunities.

Host-parasite interaction is typically characterized by significant trade-offs. Behavioral manipulation itself can impose substantial direct and opportunity costs on the parasite ^9– 11^. For example, manipulation of innate fear in infected rats is optimized and not maximized ^12^; suggesting that a dynamic balance exists for the parasite between the costs and benefits of the manipulation. From the perspective of the rat host, one of the most important trade-offs is the reproductive benefit obtained from increased attractiveness and the reproductive cost incurred by greater predation. An experimental study of this trade-off would require an ethically tenuous comparison of predation rates between control and infected animals. Another important trade-off for the host arises from the fact that pheromones produced to communicate male attractiveness are openly broadcasted, liable to be used by both the intended female audience and unintended prey or predator species. We examine this trade-off in the current study.

House mice are predated by rats. Studies have shown that around 70% of wild rats ^13, 14^ kill mice. Mouse-killing or muricide has been shown to be influenced by rearing ^15^, availability of food ^14^ social ^16^ and environmental ^17^ conditions. In addition, mice express an innate fear of rats ^18, 19^. This is characterized by the display of defensive behavior, secretion of stress hormones and activation of brain pathways dedicated to defensive behaviors. In fact, exposure to rat urine or even a recombinant rat urinary protein is sufficient to produce aversion in mice ^20, 21^, with the presence of an actual rat not being necessary. Interestingly, in the case of rats, exposure to soiled bedding is sufficient for females to infer greater attractiveness of *Toxoplasma gondii*-infected males ^5^. This suggests enhanced pheromonal production in infected males, which could result in greater kairomonal aversion in mice considering the openly broadcasted nature of urinary signals. Enhanced aversion in prey species could constitute an opportunity cost for the infected rats that need to be ‘traded-off’ with any incremental benefit of enhanced pheromone production. In light of this, we investigated whether *Toxoplasma gondii* infection increased the kairomonal valence of rat urine to its prey, mice.

## Materials and methods

### Animals

The Nanyang Technological University (IACUC number: ARF SBS/NIE-A-0106AZ) institutional animal care and use committee reviewed and approved all procedures. Twelve uninfected male Balb/c mice (7–8 weeks old, housed five/cage; (369 x 156 x 132mm; 1145T, Tecniplast, UK)) and four male Wistar rats (48 days old, housed two/cage (425 x 266 x 185mm; 1291H, Tecniplast, UK)) were obtained from the vivarium of National University of Singapore. Standard corn cob cage bedding was changed twice a week. Animals were placed on a 12 hours light-dark cycle, with temperature between 20–25°C and relative humidity ranging around 70–80%, respectively. Experiments were carried out during the light phase. Food and water was available *ad libitum*. The diet was made up of standard laboratory chow (PicoLab Rodent Diet 20, 5053) with 20% protein content. Animals from this source tested serologically negative for *Toxoplasma gondii*. This was done by incubating serum (1:1000) from the rats in 24 well plates that were coated with *Toxoplasma gondii* tachyzoites overnight at 4C. After washing the wells with PBS, polyclonal anti-rat Cy3 (1:200, Millipore, catalogue number AP189C) was added and incubated for 2 hours at room temperature. The wells were visualized under a live microscope (Nikon, 20X) under the GFP filter and Cy3 filter.

### Parasites and treatments

We used a Prugniaud strain of *Toxoplasma gondii* which has a luciferase and GFP tag (sourced from John Boothroyd, Stanford School of Medicine). Parasites were maintained as tachyzoites by passage in human foreskin fibroblast monolayers (John Boothroyd, Stanford School of Medicine). Infected fibroblasts were syringe-lysed by using a 27-gauge needle to release tachyzoites. Rats were randomly picked (a blinded person picked two rat-assigned numbers) from a population infected with tachyzoites (5 × 10 ^6^, i.p in phosphate buffered saline; n = 2) or mock-infected with sterile phosphate buffered saline (0.5ml, i.p.; n = 2). The infected rats were monitored weekly for weight loss and other signs of sickness. Fresh urine and aged urine marks were collected from rats between 6 to 8 weeks post-infection, a period know to harbor chronic infection.

### Kairomone collection

For testing response to fresh urine, rat urine was collected using metabolic cages (Harvard Apparatus). Rats were placed in this apparatus for at least 2 hours and food and water was provided. The urine was collected on the same day of testing. Rat urine contains both volatile and non-volatile substances. Urinary volatiles tend to dissipate quickly with passage of time, while non-volatiles can remain stable for weeks. In order to ascertain the contribution of non-volatiles (i.e. aged urine), a plastic Petri plate was placed in a rat cage for twenty-four hours on which the rats would urine mark. Three days (stored at RT) after removal of the Petri plate, it was used as the stimulus in the avoidance-avoidance test with mice.

### Kairomonal valence of control and infected male rat urine

Response of male mice to fresh urine obtained from control or infected rats (pooled from two rats) was studied using an avoidance-avoidance conflict paradigm. Avoidance was quantified by comparing time spent by mice in two opposing bisects of arena (76 × 9 cm; 15 cm high) during a 20 minute trial. Data on time spent was collected by automated behavioral tracking software (ANY-maze, version 4.3, Stoelting). Opposing bisects either had urine from control and infected rats (5 drops of 20 µl each, placed equidistant on absorbent paper of 5 × 7 cm). For testing response to aged urine, the three day old Petri plates from the control and infected rats were placed at terminal ends of opposing bisects. Again avoidance was quantified as described above with ANY-maze. The same set of mice were used for both behavioral assays. Experiments involving aged urine preceded the experiments with fresh urine.

### Statistics

All statistical tests were conducted using IBM SPSS software (version 20). A p value of ≤ 0.05 was considered to be significant. Student’s t-test was used to estimate statistical significance. Analysis of variance (ANOVA) was used to analyze the effect of infection status and age of urine on kairomonal communication.

## Results

Avoidance of mice in response to control or infected rat scent marks was determined using a avoidance-avoidance conflict task (n = 12 uninfected control mice). Kairomonal response to fresh urine and aged urine marks was studied separately and in sequence (aged followed by fresh marks).

ANOVA was conducted with time spent in each bisect (control or infected) and age of urine (fresh or aged) as two sources of within subject factors. ANOVA revealed a significant main effect for the infection status of scent donors (F _(1,11)_ = 8.049, *p* = 0.016). The difference between fresh and aged scent marks did not reach statistical significance (F _(1,11)_ = 0.005, *p* > 0.9). The interaction between the infection status of the donor and the age of the scent marks also did not reach statistical significance (F _(1,22)_ = 0.314, *p* > 0.5).

**Figure 1.**
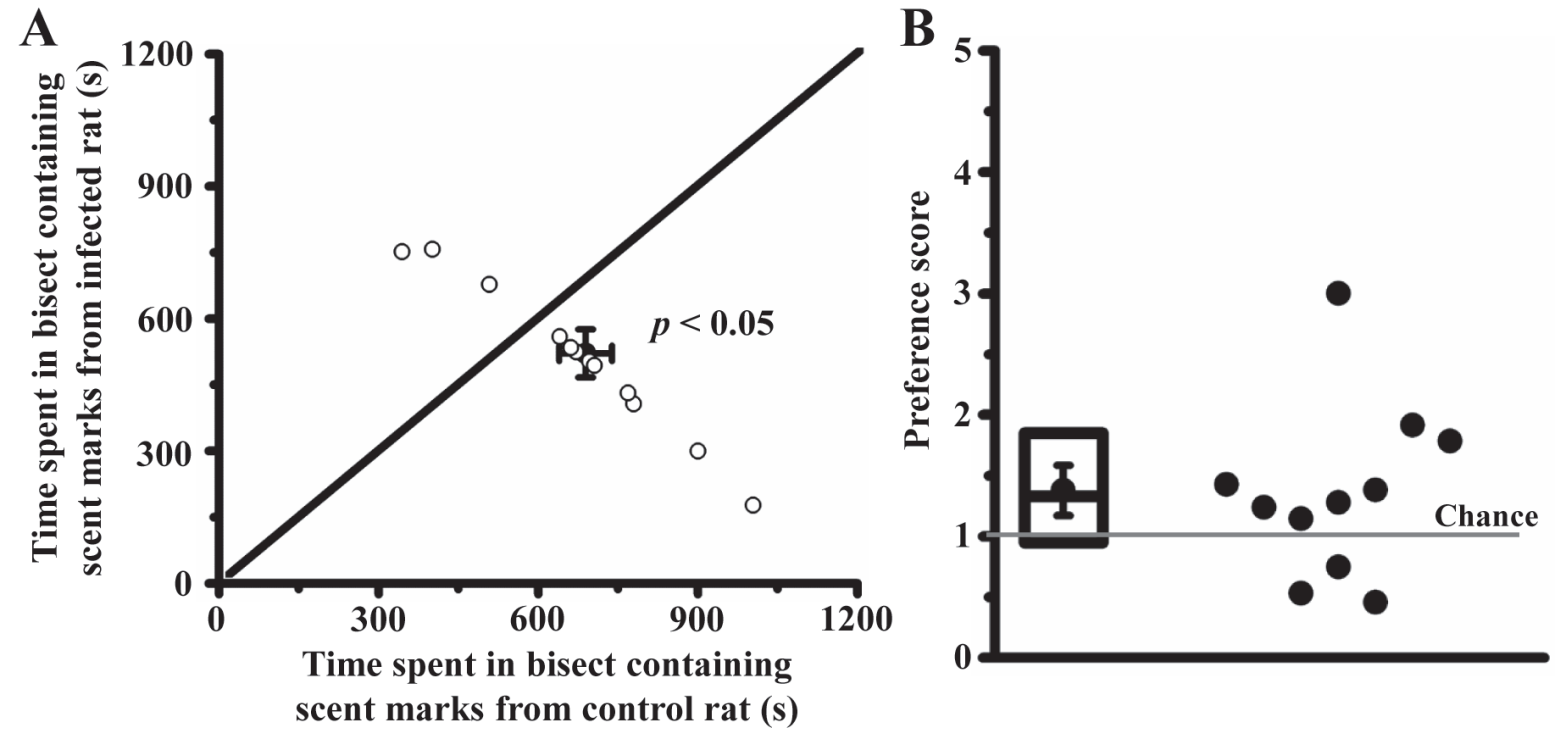
Uninfected mice avoid scent marks obtained from infected rats. Preference was quantified by comparing time spent by a mouse in two opposing bisects of an arena, with each bisect containing fresh urine from either control rats or rats infected six weeks earlier (panel **A**; trial duration = 1200 s, n = 12 mice). Ordinate and abscissa depict time spent in infected and control bisect in seconds, respectively ( *p* < 0.05, paired t-test). Mean and SEM of data used in scatter-plot are depicted by dot and whiskers. A preference score was computed for each mouse by dividing the time spent in the control bisect with time spent in the infected bisect (panel **B**; chance = 1). Each dot represents preference data from one mouse (1 outlier removed). Box plots depict median, 25 ^th^ percentile and 75 ^th^ percentile. Mean and SEM of data used in scatter-plot are depicted in dot and whiskers.

**Figure 2.**
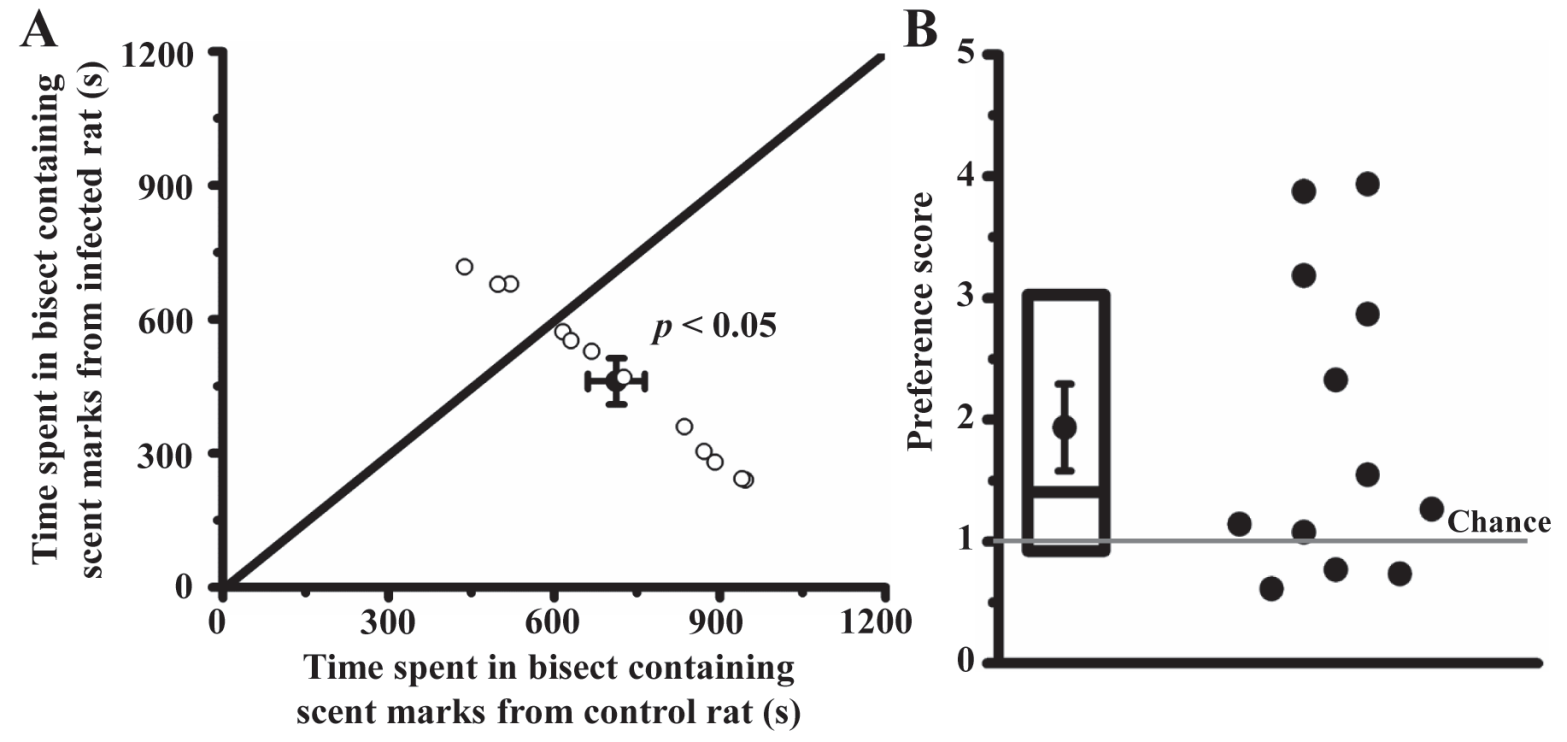
Uninfected mice avoid aged scent marks from infected rats. Preference of mice for control urine marks was retained when aged (3 days old), rather than fresh, rat urine was used. Panel **A** depicts time spent in the control and infected bisects; Panel **B** depicts preference ratio (see Figure 1 for further details on what the graphs denote).

Over a twenty-minute trial, mice spent more time in the bisect containing fresh urine from control rats (planned comparison; paired t-test: t _22_ = 2.13, *p* < 0.05; 674 ± 55 s in control bisect versus 510 ± 49 s in infected bisect; Figure 1A). A preference score of mice for control rat urine was computed for each trial by dividing the time spent in the control bisect by the time spent in the infected bisect. In 75% of trials, mice spent more time in the control bisect ( Figure 1B; mean preference score = 1.36 ± 0.22; one outlier removed). Similar results were obtained when using aged urine marks. Mice spent more time in the bisect containing aged urine marks from control rats (planned comparison; t _22_ = 3.36, *p* < 0.01; 715 ± 52 s in the control bisect versus 469 ± 51 s in the infected rat bisect; Figure 2A). Just like the fresh urine experiment, 9 out of 12 mice spent more time near the urine marks obtained from control animals ( Figure 2B; mean preference score = 1.94 ± 0.36).

Thus, fresh and aged urine marks obtained from infected rats evoke a greater kairomonal response than urine obtained from uninfected rats in mice.

Proportion of time spent by mice near urine from rats infected with Toxoplasma gondii vs urine from uninfected rats Fresh: The data set provides information on the raw times (seconds) spent by individual mice in the bisect arm with fresh uninfected rat urine (control) vs. time spent in bisect with fresh urine from rats infected with Toxoplasma gondii (total trial time: 20 minutes). The preference score of Mouse 7 was identified as an outlier and was excluded from analyses.
Aged: The data set provides information on the raw times (seconds) spent by individual mice in the bisect arm with aged (3 days) uninfected rat urine (control) vs. time spent in bisect with aged urine from rats infected with Toxoplasma gondii (total trial time: 20 minutes)

## Discussion

As a result of intense predation pressure ^13, 14^, mice have developed an innate sensitivity to rat kairomones. Rat odors evoke immediate and intense defensive behaviors in laboratory mice, coupled with activation of brain pathways typical of defensive behavior and secretion of stress hormones ^18– 20^. Here we report that urine obtained from rats infected with the parasite *Toxoplasma gondii* generate greater avoidance in mice compared to control rats. Furthermore, we demonstrate that the active ingredient involved in parasite-induced kairomonal changes is most likely non-volatile, leading us to speculate that major urinary proteins are involved. This is in agreement with a prior report that purified recombinant rat major urinary proteins can induce kairomonal aversion in mice, precluding an essential role of urinary volatiles ^20^.

Pheromones are chemical substances produced by an individual animal, which affect the behavior of conspecifics (chemical communication). Pheromones are widely used by insects to communicate danger, the presence of food or sexual receptiveness. In the mammalian world, pheromones are often used to signal dominance and to influence female mate choice ^22– 24^. Moreover, pheromones can act as kairomones when these chemical substances are received by individuals of another species ^25^. As such, kairomones are used to the detriment of the emitter and for the benefit of the receiver ^26^. This is because, once produced, pheromones are an openly broadcasted information system. Apart from the intended receivers of the same species, they can also be perceived by unintended receivers of a different species. These unintended receivers typically fall into two categories: predators and prey. Predators routinely use pheromones produced by their prey to locate their food ^27– 29^. Prey can also use this information to reduce their danger of predation ^20, 30^, although this possibility is relatively less well studied. Thus, pheromones of a species can be used as kairomones by both the prey and predator of that species.

Parasitic infections can drastically affect pheromonal production. For example, female mice typically avoid the odor of male mice infected with an array of bacteria, viruses, protozoa and nematodes ^31^. Atypically, male rats infected with the protozoan parasite *Toxoplasma gondii* produce urine that is more attractive to receptive females, suggesting enhanced pheromonal production ^5^. We have also observed that *Toxoplasma gondii* can be transmitted during sexual intercourse in rats ^5^ (see ^6, 32^ for sexual transmission in other species). It is likely that increased male attractiveness is a parasitic manipulation, aiming to increase the frequency of parasite transmission between males and females (but also see ^33^). This atypical host manipulation by *Toxoplasma gondii* opens a rather interesting trade-off for the infected host. An increase in pheromonal communication might lead to enhanced reproductive benefits for the infected male. At the same time, intra-species pheromonal communication can be co-opted by prey to initiate defensive behaviors, placing a probabilistic cost on the predator. Consistent with this trade-off, the data presented here suggests that male rats infected with *Toxoplasma gondii* suffer opportunity costs in terms of greater aversion by mice, a prey species.

## Data availability

figshare: Proportion of time spent by mice near urine from rats infected with *Toxoplasma gondii* vs urine from uninfected rats, doi: 10.6084/m9.figshare.993871 ^34^
