# Supplementary material for: Toxoplasma gondii infection enhances the kairomonal valence of rat urine
Source: F1000Res. 2014 Apr 17;3:92. [Version 1] doi: 10.12688/f1000research.3890.1 (PMC4097347; doi:10.12688/f1000research.3890.1)
Supplement: Peer review history

# Peer review: Referee response for version 1

**DOI:** 10.5256/f1000research.4166.r4524

**Published:** 2014-04-30

**Authors:** Jaroslav Flegr

**Affiliations:** 1 Department of Biology, Charles University in Prague, Prague, Czech Republic

The authors clearly showed that mice avoid the smell of urine of *Toxoplasma* infected rats more than the  smell of urine of *Toxoplasma* free rats. This effect was observed with both fresh and old urine samples, suggesting that non-volatile components of urine were responsible for the phenomenon.

Authors used the correct experimental setup and correct techniques, including the statistical methods. I see just one potentially important problem, namely a rather common problem of pseudoreplications. Authors used urine sample mixtures originated from just two *Toxoplasma* infected rats and from two *Toxoplasma* free rats. Theoretically, existence of one abnormal urine sample among four samples could give a false positive result of a study. To avoid the problem of pseudoreplications, authors should use urine sample from different rat for each mouse.

It would be interesting to test also the reaction of mice on some *Toxoplasma* infected and *Toxoplasma* free members of some non-predator species. Without doing this it could not be decided whether the mice avoided the smellier predator or the parasite. (Of course, the former explanation seems to be more probable.)

I have read this submission. I believe that I have an appropriate level of expertise to confirm that it is of an acceptable scientific standard.

# Peer review: Referee response for version 1

**DOI:** 10.5256/f1000research.4166.r4523

**Published:** 2014-04-22

**Authors:** Jian-Xu Zhang

**Affiliations:** 1 Institute of Zoology, Chinese Academy of Sciences, Beijing, China

This paper describes an interesting result - that infected rats were more aversive to prey (mice). The authors have previously reported that infected male rats produced urine marks which were more attractive to female conspecifics. They postulated that infected male rats may produce more male pheromones to attract female conspecifics, which could also act as kairomones to repel rats' prey, e.g. house mice. Although the authors did not chemically analyze the contents of the pheromonal components, at this stage their behavioral tests reliably supported their conclusion.

They should add some references to show the rat's pheromone identification.

I have read this submission. I believe that I have an appropriate level of expertise to confirm that it is of an acceptable scientific standard.
